# Retraction Note: BRD9 inhibition promotes PUMA-dependent apoptosis and augments the effect of imatinib in gastrointestinal stromal tumors

**DOI:** 10.1038/s41419-025-07756-0

**Published:** 2025-06-05

**Authors:** Jianfeng Mu, Xuezeng Sun, Zhipeng Zhao, Hao Sun, Pengda Sun

**Affiliations:** 1 Department of Gastric and Colorectal Surgery, The First Hospital of Jilin University, Changchun, China; 2 Department of Gastrointestinal Nutrition and Hernia Surgery, The Second Hospital of Jilin University, Changchun, China

Retraction Note to: *Cell Death and Disease* 10.1038/s41419-021-04186-6, published online 19 October 2021

The Editors-in-Chief have retracted this article because of concerns regarding the figures presented in this work. These concerns call into question the article’s overall scientific soundness. An investigation conducted after its publication discovered the following issues:

- Panels #3, Normal and #3, GIST in Figure 1A appear to overlap, when rotated, with Panels #1, Normal and #2, Tumor in Figure 1A in [1];
- Panel Untreated, WT in Figure 4D appears to overlap with Panel PUMA-KO, Untreated in Figure 6C in [2];
- Panel Untreated, PUMA-KO in Figure 4D appears to overlap, when rotated, with Panel WT, Untreated in Figure 6C in [2];
- Panels Untreated, WT and GSK-602, PUMA-KO in Figure 4E appear to overlap, when rotated, with Panels UT, PUMA-KD and trametinib, PUMA-KD in Figure 6D in [3].

The panels in question represent tissues taken from animals subject to different experimental conditions. The Editors-in-Chief therefore no longer have confidence in the integrity of the research presented in this article.

The authors have not replied to correspondence from the Publisher about this retraction.
